# Staged Surgical Resection of Giant Multicentric Cranial Plasma Cell Neoplasms: A Case Report

**DOI:** 10.7759/cureus.108926

**Published:** 2026-05-15

**Authors:** Hugo E Mora Moreno, Fernando Villicaña Díaz, Cesar A Durán Ramírez, Luis F Silva Pantoja, Ingrid Olvera Franco

**Affiliations:** 1 General Surgery, Hospital General Dr. Miguel Silva, Morelia, MEX; 2 Neurosurgery, Hospital General Dr. Miguel Silva, Morelia, MEX

**Keywords:** calvarial tumor, multiple myeloma, osteolytic skull lesion, plasma cell neoplasm, plasmacytoma

## Abstract

Calvarial plasma cell neoplasms are uncommon and may present as destructive osteolytic lesions, requiring careful distinction among localized plasmacytoma, multifocal plasmacytoma, and multiple myeloma. We report the case of a 45-year-old male construction worker who presented with a six-month history of a progressively enlarging, painless frontoparietal mass and a second smaller lesion in the left posterior parietal region. Neurological examination was normal. Non-contrast and contrast-enhanced CT demonstrated a large expansile osteolytic lesion centered at the cranial vertex, measuring 101 × 84 × 74 mm, with internal bony spicules, a soft-tissue component, heterogeneous enhancement, and localized mass effect on the adjacent bilateral parietal lobes. A second osteolytic lesion with similar radiological features measured 49 × 41 × 37 mm. Laboratory evaluation revealed elevated serum kappa light chains, an abnormal kappa/lambda ratio, increased IgG levels, and serum immunofixation positive for monoclonal IgG kappa. Because of the large tumor burden, surgical complexity, multicentric calvarial involvement, anticipated operative duration, potential cumulative blood loss, reconstructive planning, and airway considerations related to previous tracheal surgery, staged surgical management was performed. The dominant frontoparietal lesion was resected first through bicoronal exposure and circumferential craniectomy, followed two weeks later by resection of the second lesion and cranioplasty. Histopathological examination of both lesions demonstrated a plasma cell neoplasm, and immunohistochemistry showed CD138-positive plasma cells with kappa light-chain restriction, confirming a clonal plasma cell neoplasm. The patient recovered without neurological deficit and was referred to hematology for systemic staging. This case highlights the importance of considering plasma cell neoplasm in the differential diagnosis of multicentric destructive calvarial masses. It illustrates the potential role of staged surgical resection in selected patients with giant calvarial involvement.

## Introduction

Plasma cell neoplasms comprise a heterogeneous spectrum of clonal disorders characterized by abnormal plasma cell proliferation and monoclonal immunoglobulin production. Their classification requires integration of clinical findings, serum and urine monoclonal protein studies, bone marrow evaluation, imaging, histopathology, and immunophenotypic evidence of clonality [1]. Within this spectrum, distinguishing solitary plasmacytoma, multifocal plasmacytoma, and multiple myeloma is clinically important because these entities differ in prognosis, systemic disease burden, need for adjuvant therapy, and long-term surveillance [1,2].

Skeletal involvement is one of the most clinically relevant manifestations of plasma cell disorders. Osteolytic lesions may lead to cortical destruction, soft-tissue extension, pathological fracture, neurological compromise, or mass effect, depending on the anatomical location and extent of bone involvement [3]. Although axial skeletal involvement is more frequently emphasized in plasma cell myeloma, cranial and calvarial lesions may occasionally represent the initial or dominant clinical manifestation. In such cases, the diagnostic approach must not be limited to local tumor characterization because apparently localized cranial lesions may coexist with systemic disease or represent part of a broader plasma cell disorder [2,3].

Modern imaging plays a central role in the evaluation of plasma cell neoplasms by defining local anatomy, assessing bone destruction, identifying soft-tissue extension, and detecting additional skeletal or extramedullary lesions. Whole-body imaging, including (18F)fluorodeoxyglucose positron emission tomography/CT, is particularly useful for staging, prognostication, treatment planning, and response assessment in plasma cell disorders [4]. This is especially relevant when multiple bone lesions are present, as multicentric involvement increases the need for complete hematologic staging to differentiate multifocal plasmacytoma from multiple myeloma.

Calvarial involvement by plasma cell neoplasms is uncommon and may pose diagnostic and surgical challenges, particularly when lesions are large, destructive, multicentric, or associated with a soft-tissue component [5]. Giant calvarial masses require careful differentiation from other aggressive cranial bone tumors, including metastatic disease, primary bone tumors, and other hematologic malignancies. We report the case of a 45-year-old man with two large osteolytic calvarial masses confirmed histopathologically and immunohistochemically as clonal plasma cell neoplasms, managed with staged surgical resection and referral for definitive systemic hematologic staging.

## Case presentation

A 45-year-old male construction worker with a remote history of tracheal tumor resection and airway prosthesis placement six years earlier presented to the emergency department with a progressively enlarging mass in the frontoparietal region. Detailed histopathological information from the previous tracheal procedure was not available. The lesion had first been noticed approximately six months before presentation and had gradually increased in size. It was painless and was not associated with erythema, ulceration, warmth, drainage, or other local inflammatory changes. Approximately one month after the onset of the first lesion, the patient noticed a second painless, non-pulsatile mass in the left posterior parietal region. He denied headache, seizures, fever, night sweats, unintentional weight loss, visual disturbances, or focal neurological deficits.

**Figure 1:**
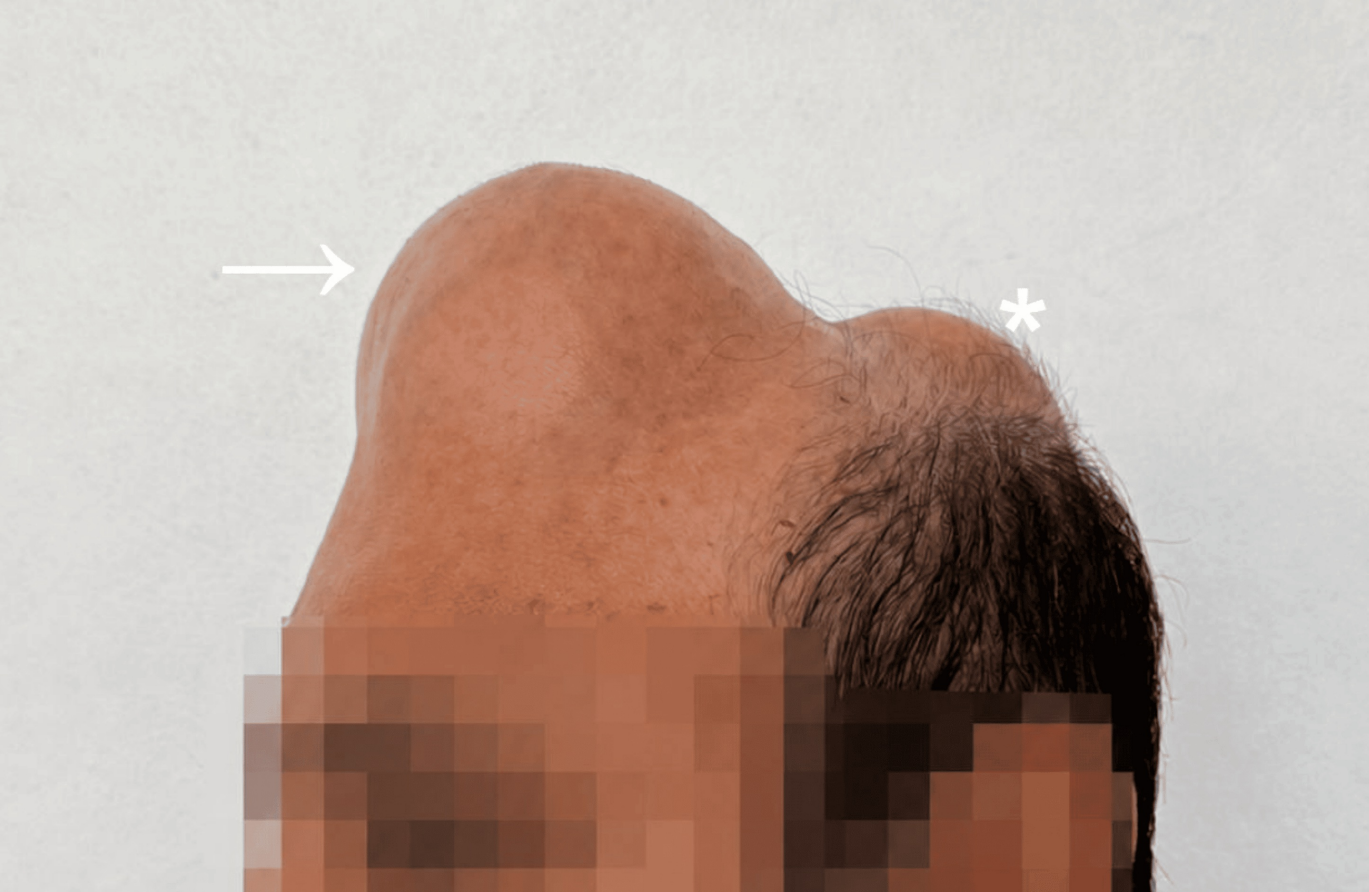
Preoperative clinical appearance of the multicentric calvarial masses Preoperative clinical photograph showing two calvarial masses. The arrow indicates the large dome-shaped frontoparietal mass, corresponding to the dominant lesion, while the asterisk marks the smaller left posterior parietal mass. The overlying skin appears intact, without evident ulceration, drainage, or inflammatory changes. The patient’s facial features were anonymized to protect privacy.

On physical examination, a firm, non-tender, immobile mass measuring approximately 13 × 12 × 10 cm was identified in the frontoparietal region. A second lesion measuring approximately 5 × 5 × 4 cm was palpated in the left posterior parietal region and showed similar clinical characteristics. The overlying skin was intact, without erythema, ulceration, warmth, or drainage. Neurological examination was normal, with a preserved level of consciousness, intact cranial nerve function, and no motor or sensory deficits. The preoperative clinical appearance of both calvarial masses is shown in Figure 1.

Selected laboratory findings relevant to the diagnostic evaluation are summarized in Table 1. Baseline hematologic parameters were preserved, with no anemia, leukocytosis, or thrombocytopenia. C-reactive protein was mildly elevated at 10.4 mg/L. Serum kappa light chains were increased to 642 mg/dL, with an abnormal kappa/lambda ratio of 6.619. Quantitative immunoglobulin testing demonstrated elevated IgG of 2,271 mg/dL and increased kappa immunoglobulin of 642 mg/dL. Serum protein immunofixation confirmed the presence of a monoclonal IgG kappa band, supporting the suspicion of a plasma cell neoplasm.

**Table 1:** Relevant laboratory findings at initial evaluation Selected laboratory findings demonstrating preserved baseline hematologic parameters, mild inflammatory activity, elevated IgG, increased serum kappa light chains, an abnormal kappa/lambda ratio, and serum immunofixation positive for a monoclonal IgG kappa band support a clonal plasma cell disorder. Reference ranges correspond to the reporting laboratory. IgG: immunoglobulin G

Parameter	Result	Reference range
Hemoglobin	15 g/dL	13-17 g/dL
Leukocytes	6.3 × 10³/µL	4.5-10 × 10³/µL
Platelets	264 × 10³/µL	150-400 × 10³/µL
C-reactive protein	10.4 mg/L	0-6 mg/L
Serum kappa light chains	642 mg/dL	155-400 mg/dL
Serum lambda light chains	97 mg/dL	83-224 mg/dL
Kappa/lambda ratio	6.619	1.29-2.61
IgG	2,271 mg/dL	710-1,540 mg/dL
Beta-2 microglobulin	2.47 mg/L	0.97-2.64 mg/L
Serum immunofixation	Monoclonal IgG kappa band	Negative

**Figure 2:**
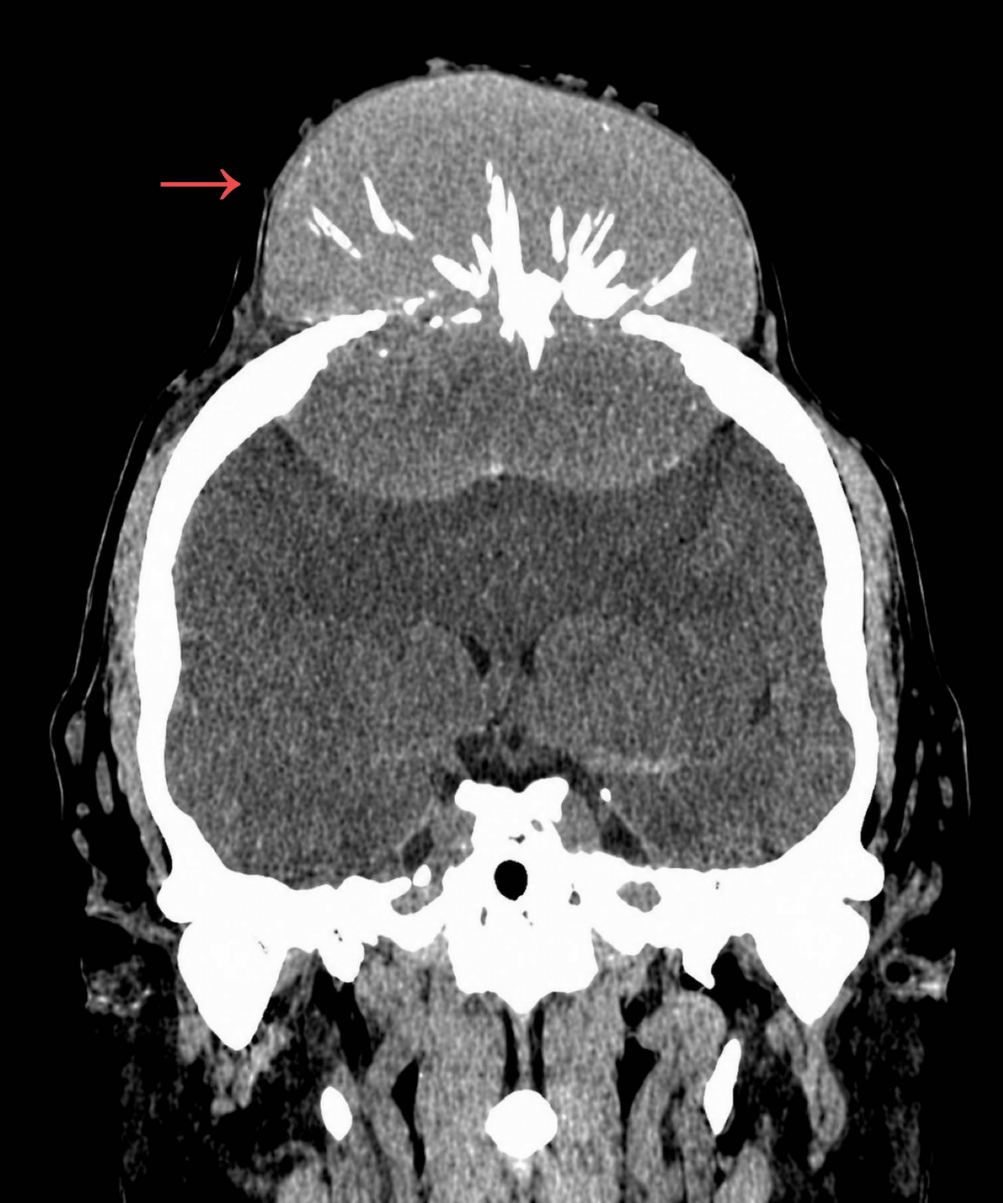
Coronal CT image showing a giant osteolytic calvarial lesion Contrast-enhanced coronal CT image demonstrating a large expansile osteolytic lesion centered at the cranial vertex, with internal bony spicules and a soft-tissue component producing local mass effect on the adjacent brain parenchyma. CT: computed tomography

**Figure 3:**
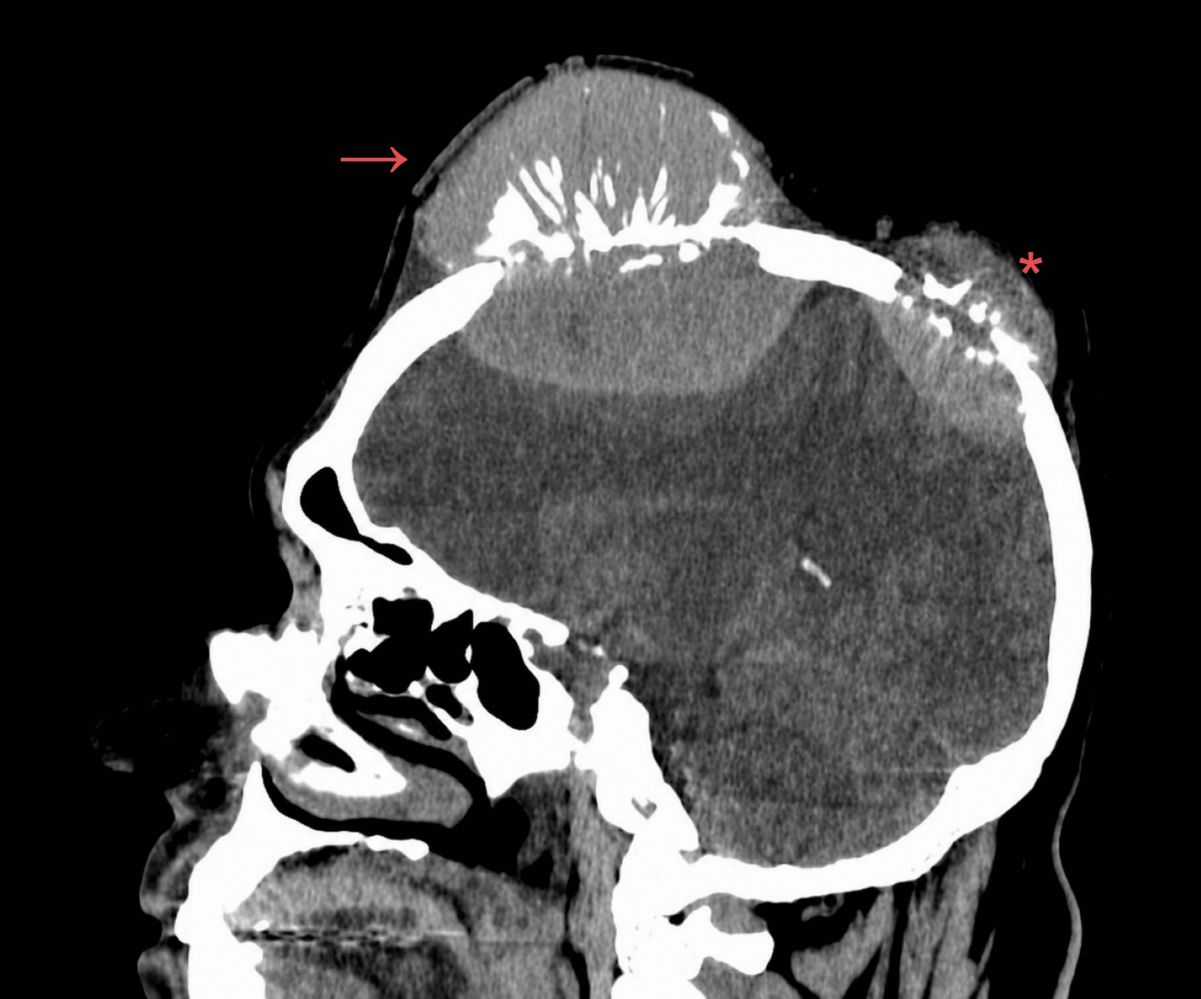
Sagittal CT image demonstrating multicentric calvarial osteolytic lesions Sagittal CT image showing two calvarial osteolytic lesions. The arrow indicates the dominant frontoparietal expansile osteolytic mass, characterized by bone destruction, soft-tissue extension, and internal bony spicules. The asterisk identifies the second smaller lesion in the left posterior parietal region, supporting multicentric calvarial involvement. CT: computed tomography

Non-contrast and contrast-enhanced cranial CT revealed a large expansile osteolytic lesion centered at the cranial vertex along the midline, measuring 101 × 84 × 74 mm. The lesion showed well-defined margins, internal bony spicules, a relatively homogeneous soft-tissue component, and heterogeneous enhancement after intravenous contrast administration. It produced a localized mass effect, compressing the adjacent bilateral parietal brain parenchyma (Figure 2). Sagittal CT reconstruction demonstrated a second, smaller lesion with similar radiological features in the left posterior parietal region, measuring 49 × 41 × 37 mm (Figure 3). Plain skull radiographs showed multiple osteolytic lesions with radiating bony trabeculae, producing a sunburst-like appearance and raising suspicion for an aggressive neoplastic bone lesion, with a plasma cell neoplasm considered within the differential diagnosis.

**Figure 4:**
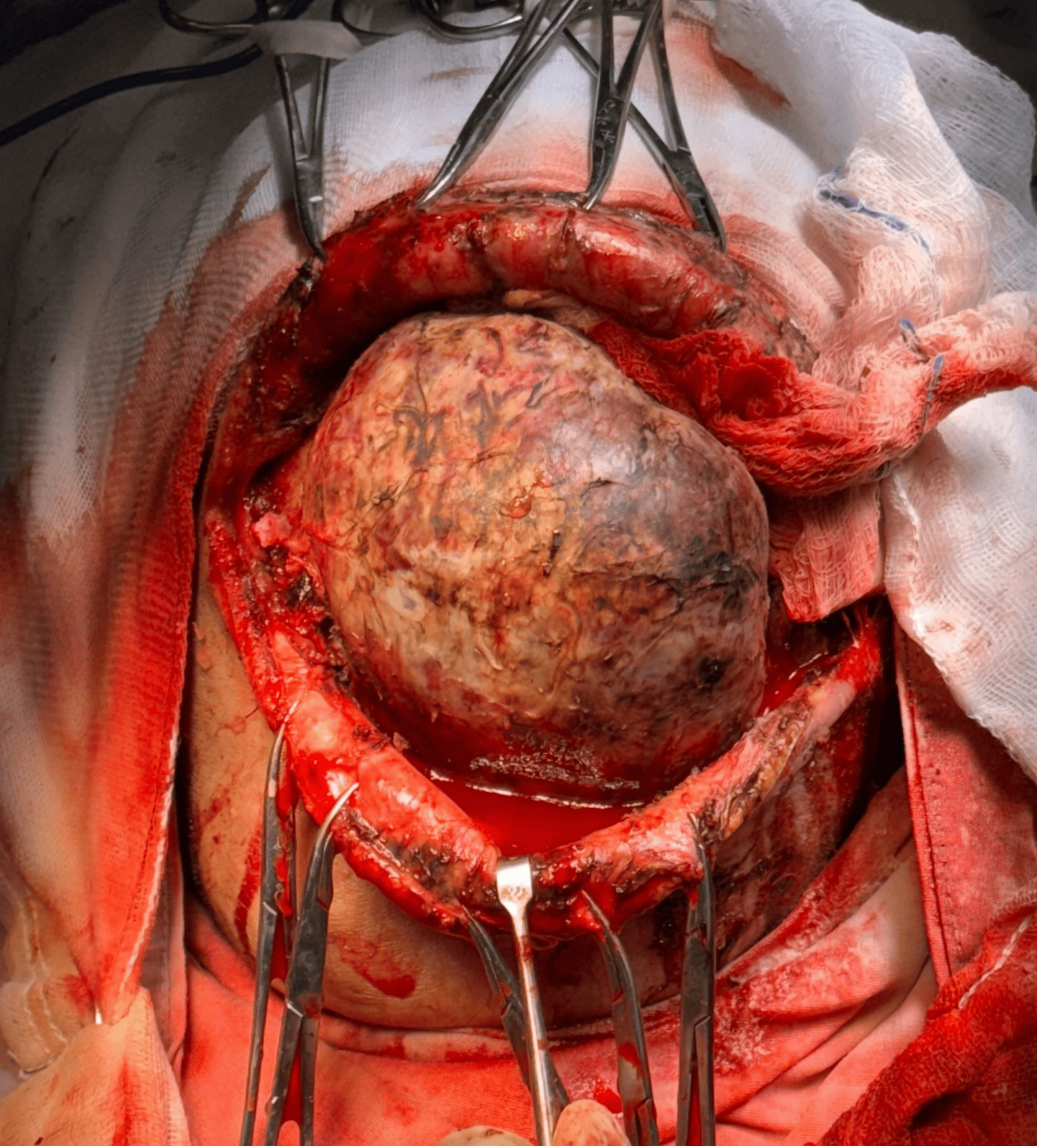
Intraoperative exposure of the dominant frontoparietal lesion Intraoperative photograph after bicoronal exposure showing the large calvarial mass before complete resection.

**Figure 5:**
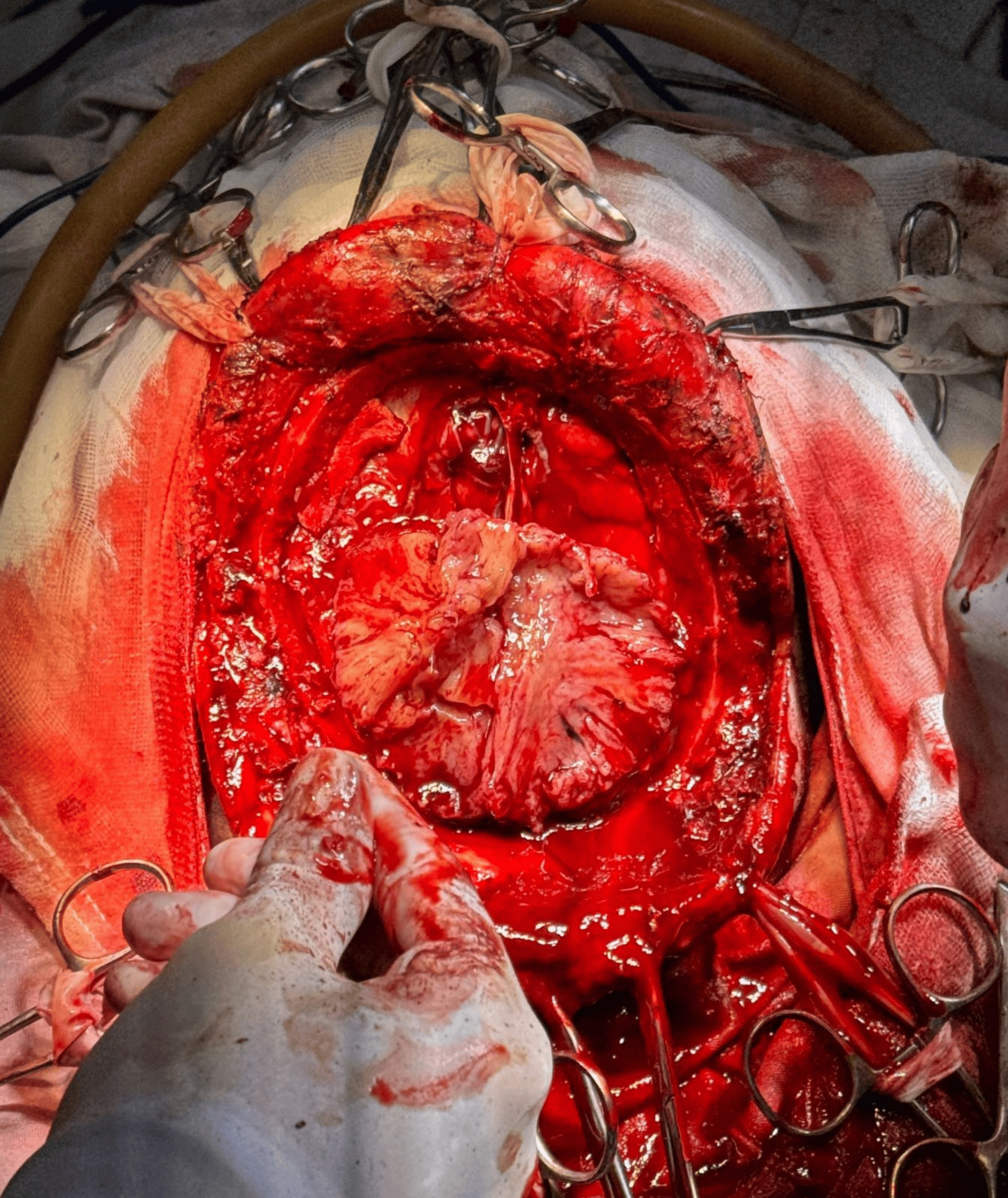
Surgical cavity after resection of the dominant calvarial tumor Intraoperative view after resection of the dominant lesion, showing the surgical cavity and preserved underlying intracranial structures. No gross dural infiltration or brain parenchymal invasion was identified.

The neurosurgery service evaluated the patient, and a staged surgical strategy was selected instead of a single-session resection because of the large tumor burden, surgical complexity, and multicentric distribution of the calvarial lesions. Additional factors supporting this approach included the anticipated operative duration, the potential for cumulative blood loss during extensive calvarial resection, the need for safer reconstructive planning, and perioperative airway considerations related to the patient’s previous tracheal surgery and airway prosthesis. Therefore, the dominant frontoparietal lesion was prioritized during the first stage. During this procedure, the frontoparietal lesion was approached through a bicoronal incision. The intraoperative exposure of the dominant lesion is shown in Figure 4. Circumferential craniectomy was performed, allowing en bloc resection of the mass. Intraoperatively, the lesion was confined to the calvarium, without gross evidence of dural infiltration or intracranial extension. The underlying brain parenchyma remained intact. The surgical cavity after resection of the dominant frontoparietal lesion is shown in Figure 5.

After surgery, the patient was admitted to the intensive care unit for postoperative monitoring and required invasive mechanical ventilation for 48 hours because of airway considerations related to his previous tracheal surgery.

**Figure 6:**
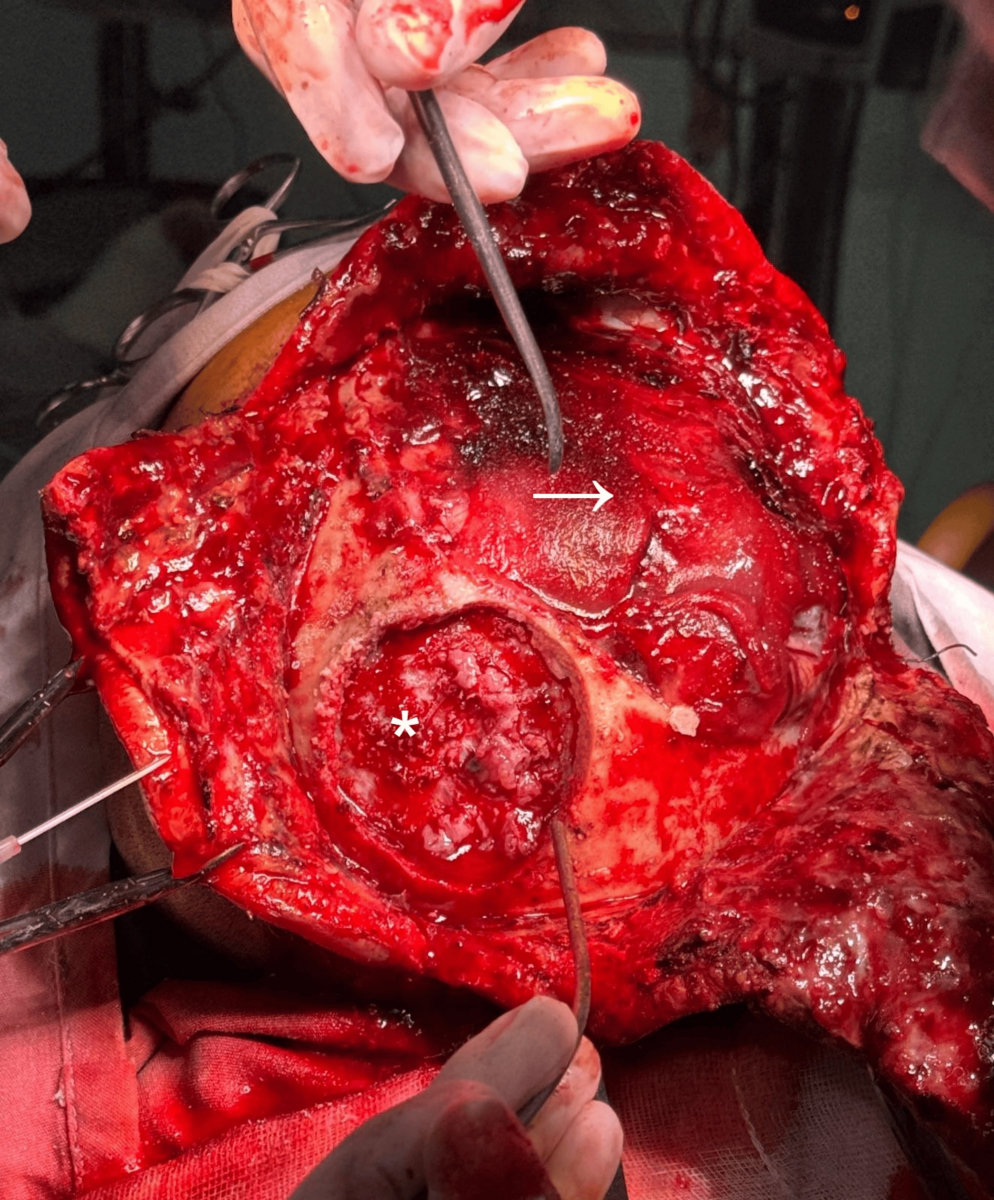
Intraoperative view after resection of both calvarial tumors Intraoperative photograph showing the calvarium after resection of both lesions. The arrow indicates the site previously occupied by the dominant frontoparietal tumor, whereas the asterisk marks the site of the second tumor in the left posterior parietal region. The image demonstrates the post-resection calvarial defects and the multicentric distribution of the lesions.

Two weeks later, a second-stage procedure was performed to resect the left posterior parietal lesion. Using a similar surgical approach, craniectomy and en bloc excision were achieved. No dural involvement or parenchymal invasion was identified intraoperatively. Cranioplasty was performed during the same procedure. An intraoperative view obtained after resection of both lesions demonstrated the calvarial defects corresponding to the dominant frontoparietal tumor and the second left posterior parietal tumor (Figure 6). The patient tolerated both procedures well, without intraoperative or immediate postoperative complications.

During the postoperative period, the patient received intravenous ceftriaxone, proton pump inhibitor therapy, and thromboprophylaxis with enoxaparin. His clinical course was uneventful, with stable vital signs, adequate wound healing, and no new neurological deficits. He was discharged on postoperative day five after the second-stage procedure, following drain removal, with instructions for outpatient follow-up.

At the outpatient follow-up visit four weeks after discharge, the patient reported adequate oral intake and denied headache, seizures, visual symptoms, or focal neurological complaints. He was alert and oriented, with no cognitive impairment or neurological deficit. Surgical sutures were removed without complication.

Histopathological examination of both resected lesions demonstrated a plasma cell neoplasm; the dominant specimen measured 12 × 8 × 6 cm. Immunohistochemical staining showed CD138-positive plasma cells with kappa light-chain restriction, supporting a clonal plasma cell neoplasm. Representative histopathological and immunohistochemical microphotographs could not be included because digital images of the slides were not available from the institutional pathology archive at the time of manuscript preparation. Therefore, the pathological diagnosis is reported based on the final signed pathology report.

Given the presence of multicentric osteolytic calvarial lesions, monoclonal IgG kappa gammopathy, elevated serum kappa light chains, and an abnormal kappa/lambda ratio, the patient was referred to the hematology service for completion of systemic staging and consideration of adjuvant therapy. Further evaluation was recommended to distinguish a multifocal plasmacytoma from multiple myeloma, including bone marrow aspiration and biopsy, urine protein electrophoresis with immunofixation, whole-body imaging, and a complete assessment of myeloma-defining events and end-organ damage, including hypercalcemia, renal impairment, anemia, and additional bone lesions. At the time of manuscript preparation, the results of post-referral hematologic staging studies were not available to the authors; therefore, the final systemic classification could not be established.

## Discussion

Plasma cell neoplasms represent a diagnostic spectrum ranging from localized plasmacytoma to systemic multiple myeloma, and accurate classification requires correlation among histopathology, immunohistochemistry, serum and urine monoclonal protein studies, bone marrow assessment, and whole-body imaging [6,7]. In the present case, the diagnosis of a clonal plasma cell neoplasm was supported by histopathological examination of both resected lesions, CD138-positive plasma cells with kappa light-chain restriction, monoclonal IgG kappa gammopathy, elevated serum kappa light chains, and an abnormal kappa/lambda ratio. However, the presence of multicentric calvarial osteolytic lesions makes definitive systemic classification essential, as multifocal plasmacytoma and multiple myeloma may overlap clinically and radiologically. Current diagnostic frameworks emphasize that bone marrow aspiration and biopsy, evaluation for myeloma-defining events, assessment of serum and urine monoclonal protein levels, and systemic imaging are necessary to distinguish localized from systemic disease [6,7]. In this patient, post-referral hematologic staging results were not available at the time of manuscript preparation, and the final systemic classification, therefore, remained pending.

The immunohistochemical profile is particularly important in cases presenting as destructive bone masses, because the radiological differential diagnosis of calvarial osteolytic lesions is broad and includes metastatic carcinoma, primary bone tumors, lymphoma, vascular tumors, and other hematologic malignancies. CD138 expression supports plasma cell differentiation, while light-chain restriction confirms clonality, helping to separate reactive plasmacytic infiltrates from neoplastic plasma cell proliferation [8]. In our patient, immunohistochemistry confirmed a clonal plasma cell neoplasm, but this pathological confirmation should be interpreted as complementary to rather than a replacement for systemic staging. Therefore, the most appropriate interpretation at the time of surgical discharge was a histopathologically and immunohistochemically confirmed plasma cell neoplasm with multicentric calvarial involvement, pending definitive hematologic classification. A limitation of this report is the absence of representative histopathology and immunohistochemistry microphotographs, as digital slide images were not available for inclusion in the manuscript; however, the diagnosis was based on the final pathology report documenting a plasma cell neoplasm with CD138 expression and kappa light-chain restriction.

Solitary plasmacytoma is classically defined as a localized clonal plasma cell tumor without evidence of systemic multiple myeloma. Still, progression to multiple myeloma remains a major concern, particularly in patients with bone involvement or abnormal monoclonal protein studies [9,10]. Large contemporary series and reviews emphasize the importance of careful staging and long-term surveillance because an initially localized presentation may later evolve into systemic disease [9,10]. In the present case, the multicentric distribution of the calvarial lesions and the abnormal serum monoclonal profile increased concern for a systemic plasma cell disorder. For this reason, the patient was referred to hematology for completion of staging, including bone marrow evaluation; urine protein electrophoresis with immunofixation; assessment of hypercalcemia, renal impairment, anemia, and additional bone lesions; and whole-body imaging.

The epidemiology of plasma cell neoplasms varies according to disease subtype, with solitary plasmacytoma, extramedullary plasmacytoma, and plasma cell myeloma representing distinct but related entities [11]. Extramedullary disease and atypical skeletal presentations are clinically important because they may be associated with diagnostic delays, unusual local symptoms, and more complex treatment decisions [12,13]. Although calvarial involvement is anatomically skeletal rather than purely extramedullary, giant calvarial masses with soft-tissue extension share several practical challenges with extramedullary presentations, including local mass effect, cosmetic deformity, potential involvement of adjacent neurovascular structures, and the need for multidisciplinary management.

Imaging is central in the evaluation of plasma cell neoplasms because it defines local tumor anatomy, characterizes bone destruction, identifies soft-tissue extension, and detects additional lesions that may alter staging and treatment [14]. In this case, cranial CT demonstrated two expansile osteolytic calvarial lesions, including a dominant frontoparietal mass with internal bony spicules, heterogeneous enhancement, and compression of the adjacent parietal lobes. These findings established the extent of calvarial destruction and helped guide the staged surgical strategy. However, local cranial imaging alone is insufficient for the complete classification of plasma cell disorders. Whole-body imaging, including positron emission tomography/CT when available, is useful for identifying additional skeletal or extramedullary disease and for distinguishing apparently localized plasmacytoma from systemic involvement [14,15].

Cranial and skull-base plasmacytomas are uncommon, and their clinical presentation depends on location, size, and involvement of adjacent structures [16]. Reported intracranial and skull-base cases may present with headache, cranial nerve deficits, visual symptoms, or other neurological manifestations, whereas calvarial lesions may initially appear as painless, enlarging scalp masses [16,17]. Our patient had a notable presentation because both lesions were clinically evident as painless calvarial masses. Yet the neurological examination remained normal despite the large size of the dominant lesion and the local mass effect on imaging. This highlights that substantial calvarial destruction and intracranial compression can occur without early neurological deficits, especially when tumor growth is gradual.

The management of cranial plasma cell neoplasms should be individualized according to tumor location, symptoms, tumor size, local extension, diagnostic certainty, and systemic staging. Plasma cell tumors are generally radiosensitive, and radiotherapy plays an important role in local disease control, particularly for solitary plasmacytoma and selected head-and-neck presentations [18]. Nevertheless, surgery may be appropriate when there is significant mass effect, diagnostic uncertainty, extensive bone destruction, cosmetic deformity, risk to adjacent intracranial structures, or the need for tissue diagnosis and local control. In the present case, staged surgical resection was selected because of the large size of the dominant frontoparietal lesion, the presence of a second posterior parietal lesion, the complexity of reconstruction, and the need to safely manage multicentric calvarial disease.

The staged approach used in this patient allowed controlled resection of the dominant lesion first, followed by resection of the second lesion and cranioplasty during the subsequent procedure. In this case, staging was chosen to reduce the cumulative burden of a single prolonged operation, given the size of the dominant lesion, the presence of a second calvarial tumor, the potential for relevant blood loss during extensive calvarial resection, the need for reconstructive planning, and perioperative airway considerations related to the patient’s previous tracheal surgery and airway prosthesis. This strategy may reduce operative burden in selected patients with large or multicentric calvarial tumors, particularly when airway considerations, anticipated blood loss, reconstructive planning, or postoperative monitoring are relevant. In our case, both procedures were completed without intraoperative or immediate postoperative complications, and the patient was discharged without new neurological deficits. Although surgical resection provided local control and tissue confirmation, definitive treatment planning required hematologic evaluation, as systemic therapy, radiotherapy, or surveillance may be indicated depending on the final classification and staging [18,19].

The role of adjuvant systemic therapy in plasmacytoma remains an area of ongoing clinical interest. Some studies have evaluated systemic treatment strategies to prevent progression from solitary bone plasmacytoma to multiple myeloma. Still, treatment decisions must be individualized and guided by hematology after complete staging [19]. In this case, the abnormal monoclonal profile and multicentric bone involvement made referral for systemic evaluation particularly important. Therefore, the surgical outcome should be viewed as one component of multidisciplinary care rather than definitive oncologic management.

This case has several educational implications. First, plasma cell neoplasm should be included in the differential diagnosis of destructive calvarial masses, especially when lesions are osteolytic, expansile, multicentric, or associated with monoclonal gammopathy. Second, immunohistochemical confirmation of clonality is essential but, by itself, does not establish whether the disease is localized or systemic. Third, multicentric calvarial involvement should prompt complete hematologic staging to differentiate multifocal plasmacytoma from multiple myeloma. Finally, staged surgical resection can be a reasonable option in selected patients with giant calvarial lesions when local mass effect, diagnostic confirmation, bone destruction, and reconstructive considerations are present.

## Conclusions

Multicentric calvarial plasma cell neoplasms are uncommon and may present as progressively enlarging, painless cranial masses with extensive osteolytic bone destruction and limited or absent neurological symptoms. This case highlights the importance of including plasma cell neoplasm in the differential diagnosis of destructive calvarial lesions, particularly when imaging demonstrates multicentric osteolytic involvement and laboratory studies reveal monoclonal gammopathy. Histopathological examination and immunohistochemical confirmation of plasma cell clonality are essential for diagnosis; however, definitive classification requires complete systemic hematologic staging to distinguish multifocal plasmacytoma from multiple myeloma. In selected patients with giant calvarial lesions, staged surgical resection can provide local disease control, relieve mass effect, allow tissue diagnosis, and facilitate subsequent reconstructive planning. Multidisciplinary follow-up with hematology is essential for completing staging and determining the need for adjuvant therapy or systemic treatment.
